# An experimental study on tolerance to hypoxia in tardigrades

**DOI:** 10.3389/fphys.2023.1249773

**Published:** 2023-09-05

**Authors:** Philip Hagelbäck, K. Ingemar Jönsson

**Affiliations:** Department of Environmental Science, Kristianstad University, Kristianstad, Sweden

**Keywords:** hypoxia, anoxybiosis, tardigrades, *Richtersius* cf. *coronifer*, *Hypsibius exemplaris*

## Abstract

**Introduction:** Tardigrades are small aquatic invertebrates with well documented tolerance to several environmental stresses, including desiccation, low temperature, and radiation, and an ability to survive long periods in a cryptobiotic state under arrested metabolism. Many tardigrade populations live in habitats where temporary exposure to hypoxia is expected, e.g., benthic layers or substrates that regularly undergo desiccation, but tolerance to hypoxia has so far not been thoroughly investigated in tardigrades.

**Method:** We studied the response to exposure for hypoxia (<1 ppm) during 1–24 h in two tardigrade species, *Richtersius* cf. *coronifer* and *Hypsibius exemplaris*. The animals were exposed to hypoxia in their hydrated active state.

**Results:** Survival was high in both species after the shortest exposures to hypoxia but tended to decline with longer exposures, with almost complete failure to recover after 24 h in hypoxia. *R.* cf. *coronifer* tended to be more tolerant than *H. exemplaris*. When oxygen level was gradually reduced from 8 to 1 ppm, behavioral responses in terms of irregular body movements were first observed at 3–4 ppm.

**Discussion:** The study shows that both limno-terrestrial and freshwater tardigrades are able to recover after exposure to severe hypoxia, but only exposure for relatively short periods of time. It also indicates that tardigrade species have different sensitivity and response patterns to exposure to hypoxia. These results will hopefully encourage more studies on how tardigrades are affected by and respond to hypoxic conditions.

## 1 Introduction

Tardigrades are micro-metazoans inhabiting a wide range of environments and micro-habitats around the world, from the poles to the deep sea, and including both permanently aquatic conditions and terrestrial habitats that for shorter or longer periods are deprived of moisture (Nelson et al., 2018). The phylum Tardigrada currently includes more than 1,400 species (Degma and Guidetti, 2023). Despite a large diversity in habitat choice all tardigrades are essentially aquatic animals and need to be surrounded by water to be active. Nevertheless, tardigrades living in terrestrial habitats have evolved adaptations to survive a complete deprivation of body water, a state at which metabolism necessarily ceases and the animal enters an ametabolic state of cryptobiosis (e.g., Keilin, 1959; Wright et al., 1992; Wright, 2001; Møbjerg et al., 2011). Cryptobiosis cannot only be induced by desiccation (anhydrobiosis), but also by cold (cryobiosis), osmotic pressure (osmobiosis), and oxygen deficiency (anoxybiosis) (Keilin, 1959). Among these categories, anhydrobiosis (e.g., Crowe, 1971; Rebecchi et al., 2007; Welnicz et al., 2011; Schill and Hengherr, 2018; Arakawa, 2022) is by far the most studied phenomenon followed by cryobiosis (e.g., Ramløv and Westh, 1992; Guidetti et al., 2011; Hengherr and Schill, 2018; Møbjerg et al., 2022) and osmobiosis (e.g., Halberg et al., 2009; Heidemann et al., 2016; Emdee et al., 2023). Reports of tolerance to low oxygen conditions (hypoxia) in tardigrades are very scarce and mainly restricted to anecdotal observations describing that tardigrades may enter an asphyctic state in response to oxygen deficiency and may survive in this state for a few days (e.g., Ramazzotti and Maucci, 1983; Nelson et al., 2015). Crowe and Higgins (1967) reported that the ability of desiccated (anhydrobiotic) tardigrades to rehydrate and resume activity tended to decline with oxygen levels below 5 ppm. Also, two species of marine tardigrades (*Dipodarctus subterraneus* and *Tanarctus ramazzottii*) were reported from an outlet area in the Black Sea at a depth of 88–250 m (Kharkevych and Sergeeva, 2013), where oxygen levels at 88–122 m were estimated at 0.12–0.17 ppm (mg/L), suggesting that these populations of tardigrades live under more or less anoxic conditions. Several other invertebrate metazoa have been found in this area of permanent anoxia, with Nematoda, Harpacticoida and Polychaeta being the most abundant (Sergeeva and Mazlumyan, 2015). Some of the adaptations of deep-sea meiobenthos are reviewed in Zeppilli et al. (2018), but the adaptations of tardigrades in these environments are unknown. In contrast to anhydrobiosis and osmobiosis, which is connected to dehydration and contraction of the body into a “tun-state” (Møbjerg and Neves, 2021), the response to hypoxia in tardigrades is immobilisation and inflation of the body with the eight legs protruding from the body trunk. This behavioral response has been interpreted as resulting from a lack of osmoregulatory control (Nelson et al., 2015).

So far, no specific studies evaluating the tolerance to hypoxia in hydrated tardigrades have been reported, and there are also no studies evaluating if the metabolism of tardigrades comes to a halt when oxygen are depleted and the animals enter the asphyctic state. The existence of a cryptobiotic state *sensu stricto* induced by low oxygen levels under hydrated conditions has also been challenged (Wright et al., 1992; Clegg, 2001) and remains to be verified.

Given that the ability to enter cryptobiosis in response to various environmental agents represent adaptations for survival, species adapted to different environmental conditional are also expected to exhibit different levels of tolerance. In line with this, previous studies have found inter-specific differences among tardigrade species in tolerance to desiccation (Wright, 1989; Jönsson et al., 2001; Rebecchi et al., 2006), cold (Hengherr et al., 2009), and osmoregulation (Møbjerg et al., 2011). Such differences may also be expected with respect to tolerance to hypoxia and the ability to enter anoxybiosis, but no such comparative data are available.

Here we present the first experimental study on tolerance to hypoxia in tardigrades, evaluating the response to exposure to hypoxia for time periods up to 24 h in two different species of tardigrades.

## 2 Materials and methods

### 2.1 Tardigrade species used in the study

We used the two eutardigrade species *Hypsibius exemplaris* and *Richtersius* cf. *coronifer*, the former a freshwater species and the latter a limno-terrestrial species inhabiting mosses. Previous studies have documented a high tolerance to both desiccation and freezing in *R.* cf. *coronifer* (e.g., Ramløv and Westh, 1992; Jönsson et al., 2001; Jönsson et al., 2008; Persson et al., 2011), while *H. exemplaris* is more sensitive to these environmental agents (e.g., Wright, 1989; Bertolani et al., 2004; Horikawa et al., 2013; Poprawa et al., 2022).

Specimens of *H. exemplaris* were obtained from a population cultured in the lab, fed on a diet of chlorella algae and kept at 15°C. This population originates from a British strain that was previously named *Hypsibius dujardini* but has been redescribed as *H. exemplaris* (Gąsiorek et al., 2018). The habitat of the original collection was the benthic layer of a pond.

*R.* cf. *coronifer* was extracted from moss growing on carbonated rock at Ölands Alvar in south-eastern Sweden (see description of habitat in Jönsson et al., 2001) using the Baermann funnel method (Czerneková et al., 2018). The funnels were set up using deionized water (EASYpure^®^ RF, m. 07033, Barnstead/Thermolyne, Dubuque, IA, United States) in the day before the experiment and left for 12–13 h after which the extracted animals were left to acclimatize in new water for 2 h.

For both species only normally moving adult animals of medium and large size were selected for use in the experiment. In total, 630 specimens of each tardigrade species were used in the main experiment, including hypoxia-exposed specimens + controls (see Section 2.3.1), and 50 specimens in the experiment with increased hypoxia (see Section 2.3.2). The body size of individual specimens was not measured in this study but the mean size of adult *R.* cf. *coronifer* has been reported as 645 μm (Czernekova and Jönsson, 2016) and of *H*. *exemplaris* as 232 μm (Gąsiorek et al., 2018). Both species consist of females with parthenogenetic reproduction.

### 2.2 Method for creating a hypoxic environment

Based on the evaluation of methods for removing dissolved oxygen from water by Butler et al. (1994) we used high purity nitrogen gas (Nitrogen Instruments 5.0, ≥99,999%) to create hypoxic conditions. Deionized water (see Section 2.1) was used in all experiments. The nitrogen flow was set to 65 mL/min and was chosen to create a steady but not to vigorous purging. The flow was measured with a Porter Instrument B-125-20 flowmeter. To facilitate the spread of nitrogen in the water, an aquarium diffusion stone was attached to the end of the tube. The lowest concentrations of dissolved oxygen obtained in this study was 0.2–0.3 ppm after purging with nitrogen, which is in line with the study by Butler et al. (1994). Dissolved oxygen in water was measured using a HACH HQ40d multimeter with a LDO101 probe (HACH, Colorado, United States).

### 2.3 Experimental design

#### 2.3.1 Exposure to hypoxia for different time periods

The general design of this experiment was to expose hydrated active tardigrades to hypoxia during different time periods (1, 6, 12, 18, and 24 h) and evaluate the conditions of the animals immediately after the exposures and approximately 10 h after exposure.

In each exposure trial, seven replicate samples were used, each of them with 10 animals, contained in 100 mL Duran^®^ laboratory glass bottles (Schott, Mainz, Germany) filled with 80 mL of deionized water. The seven samples exposed to hypoxia were connected serially to the nitrogen gas cylinder via plastic tubes, and the bottles were sealed with airtight rubber stoppers with double tubing allowing inflow and outflow of nitrogen gas (see Supplementary Figure S3).

In all except the 1 h exposure trial seven control samples were used, each with 10 animals. It was considered unnecessary to use controls in the 1 h trial due to the short time span. The control samples were not exposed to hypoxia, but otherwise kept under similar conditions. Tardigrades were transferred to the bottles prior to the start of the introduction of the nitrogen gas, using a Pasteur pipette. For each exposure time period tested, a new set of tardigrades were used. The same experiments were performed for *R*. cf. *coronifer* and *H*. *exemplaris*, but in separate trials.

Since the animals were placed in the bottles before the nitrogen flow was initiated, the effective exposure time at the lowest oxygen level in all trials is shorter than the reported exposure times, and relatively more so in the short-time than in the long-time exposures. The rate at which oxygen level declined in our experiment was not measured, but pre-experimental tests showed that oxygen levels in the lowest range had been reached after approx. 25 min. Butler et al. (1994) showed that rate of deoxygenation using the same method (but with larger water volume and higher gas flow) is more rapid at the beginning and slows down as the water gets saturated with nitrogen. The animals in our study therefore may well have experienced low enough oxygen levels to enter an asphyctic state within 10–15 min after the start (see Result Section 3.4). By allowing a gradual change in the oxygen conditions, both towards low oxygen in the initial phase of the trial and towards restored oxygen after the trial (see below), the animals were allowed to make physiological adjustments in response to gradually changed oxygen conditions.

The oxygen levels in each of the 14 bottles (7 treatments +7 controls) were measured prior to each experiment, and when the experiment had been running for the set amount of time the nitrogen flow was stopped and oxygen levels were again measured. The oxygen level of the deionized water before any treatment was on average 8.43 ppm (SD = 0.10; estimated as the mean value based on the means for all experiments trials). The mean (SD) level of oxygen at the end of the 1, 6, 12, 18, and 24 h trials was 0.66 (0.11), 0.42 (0.090), 0.66 (0.037), 0.44 (0.10), and 0.52 (0.097) ppm for *H. exemplaris*, respectively, and 0.75 (0.090), 0.38 (0.064), 0.47 (0.14), 0.23 (0.044), and 0.31 (0.091) ppm for *R.* cf. *coronifer*. The experiment was performed in a laboratory with natural daylight and a temperature of 20–22°C.

For ease of observation, the animals from each bottle were directly transferred together with the 80 mL water used in the experiment to a 100 mL plastic cup (Kebolab AB, 40 mm high, 65 mm upper diam.) without cap after the hypoxia treatment. Thus, no new water was added but instead the low oxygen water was allowed to reoxygenate naturally from oxygen in the air. The water surface diameter in the cup with 80 mL water was 62 mm and the water depth 30 mm.

#### 2.3.2 Exposure to increased levels of hypoxia

To investigate how reduction of oxygen level affected the tardigrades and at what level of hypoxia effects on the animals appeared we performed a separate experiment. We used the same two species and five replicate samples with 10 animals for each species. The samples were kept in 100 mL plastic cups (see Section 2.3.1) filled with 80 mL deionized water, and the replicate samples were treated one by one (thus not in parallel). No controls were used in this experiment.

At the start, the oxygen level in each replicate cup were measured, and oxygen level was then reduced by purging nitrogen gas using a tube from the nitrogen tank and a diffusion stone, as described in Section 2.2. Oxygen level was measured continuously, and tardigrade behavior was recorded stepwise at every 1 ppm, beginning at 8 ppm and with the final recording at 1 ppm.

#### 2.3.3 Rate of natural reoxygenation

To document the rate of reoxygenation after the samples were transferred from the hypoxic conditions to the cup with exposure to open air in the laboratory, a separate test was performed. Seven 100 mL plastic cups (see Section 2.3.1) were filled with 80 mL of deionized water, and nitrogen gas was then purged to lower the oxygen level to 0.3 ppm. The seven cups were then left without cover and allowed to reoxygenate from the surrounding air and the oxygen levels were measured every 0.5 h for 3 h.

#### 2.3.4 Recording of animal activity

**FIGURE 1:**
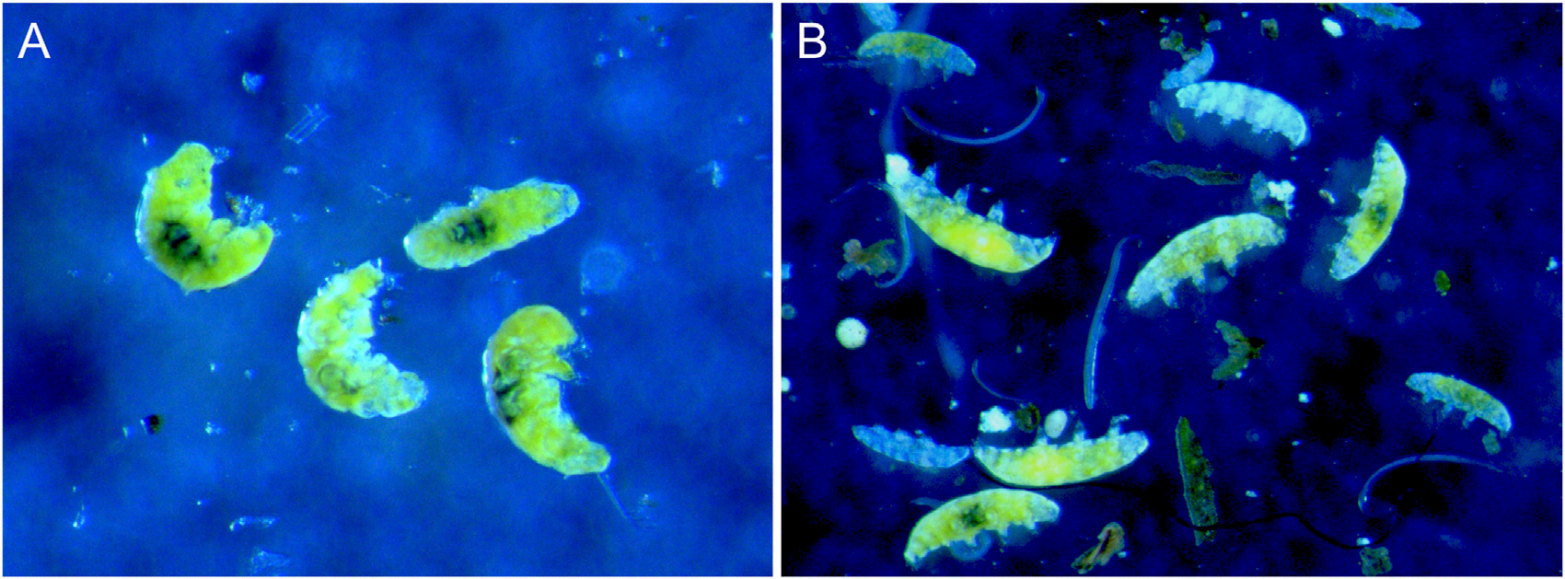
*Richtersius* cf. *coronifer* with **(A)** normal movements under normal oxygen levels in water, and **(B)** during hypoxia with inflated (asphyctic) bodies under low oxygen. Photo: K.I. Jönsson.

After the hypoxia exposure and transfer of animals to 100 mL plastic cup (see Section 2.3.1) the behavior of individual animals was recorded twice; immediately and after 10 h. Observations were made with an Olympus SZX9 stereo microscope. We considered 10 h as likely sufficient for asphyctic animal to recover and return to a state with regular movements based on personal experience with tardigrades exposed to hypoxia and recovering within half an hour when provided oxygenated water. However, knowledge on how recovery time may vary with exposure to different levels of hypoxia are currently lacking. We originally classified tardigrade behavior as “Regular movement,” “Irregular movement,” “Hypoxic” and “Dead”. The animals were classified as having regular movement when they were moving with normal unimpeded leg movements. Irregular movements represent animals with irregular or slow leg movements. Animals identified as hypoxic were in an immobile asphyctic state with inflated and stretched out bodies (interpreted as a response to oxygen deficiency), while animals identified as dead were also usually stretched out but with body content disintegrated. Although we initially tried to distinguish between these two immobile categories, and dead animals are usually easy to identify from their disintegrated body contents, the status of apparently asphyctic animals is more problematic with respect to the living status. In the analyses we therefore put together animals classified as hypoxic and dead into the same category and used three behavioral categories: regular movement (RM), irregular movement (IM), and no movement (NM). Figure 1 shows the appearance of tardigrades with regular movements and tardigrades in an asphyctic state with no movements induced by low oxygen.

#### 2.3.5 Statistical analyses

Statistical analyses were made using IBM SPSS Statistics (v. 24). The distribution of data was evaluated from histograms and Q-Q plots using unstandardized residuals from a univariate GLM analysis, with exposure as independent variable and behavioral response categories as dependent variables. Since residuals were found to be non-normally distributed, non-parametric tests were used in all analyses. For analyses of differences between treatment groups the Kruskal–Wallis Analysis of Variance test within the Nonparametric tests/Independent samples module of SPSS was used. This module also provided pairwise comparisons between groups, based on Dunn´s *post hoc* test. Treatment groups were considered statistically different when *p* < 0.05. The standard uncorrected *p*-values were used as the base for interpreting the results, but in pairwise post hoc tests the Bonferroni-adjusted values are also presented for comparison. The adjusted *p*-values were not used as the Bonferroni correction has been criticized to warp results, and while decreasing the risk of type I error, it also increases the risk of type II errors (Perneger, 1998; Armstrong, 2014).

For control samples, no statistical differences were found among controls for the different time categories (within the three behavioral groups), neither for *R.* cf. *coronifer* or *H*. *exemplaris*, and within each species the control data were therefore pooled.

## 3 Results

### 3.1 *Richtersius* cf. *coronifer*

**FIGURE 2:**
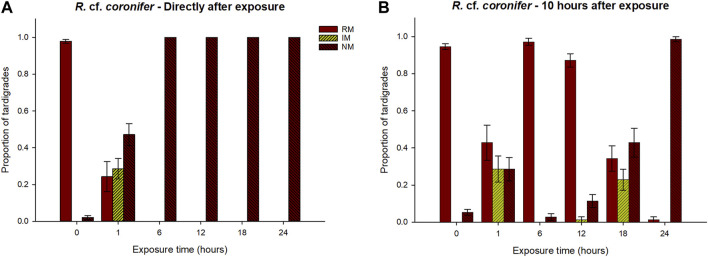
Responses of *R.* cf. *coronifer* to exposure of hypoxic conditions for different periods of time (0–24 h), in terms of proportions of tardigrades recorded in the three behavioral categories regular movement (RM), irregular movement (IM), and no movement (NM). 0 represents the control group that was not exposed to hypoxia. Panel **(A)** shows the results at the first check directly after exposure, while panel **(B)** shows the results 10 h after the exposure. Error bars represent 1 standard error from 7 replicate samples, each with 10 individual tardigrades. Statistical data for overall comparisons between exposure groups: Panel **(A)**. RM: χ^2^ = 58.0, *p* < 0.001. IM: χ^2^ = 52.1, *p* < 0.001. NM: χ^2^ = 59.4, *p* < 0.001. Panel **(B)**. RM: χ^2^ = 47.4, *p* < 0.001. IM: χ^2^ = 47.4, *p* < 0.001. NM: χ^2^ = 41.8, *p* < 0.001. Degrees of freedom = 5 in all comparisons.

The proportion of animals of *R.* cf. *coronifer* in the three behavioral categories after exposures to hypoxic conditions for different periods of time is shown in Figure 2 (for original data see Supplementary Table S1). In the first check directly after the exposure, 100% of the tardigrades were in a no movement (NM) state after 6 h, 12 h, 18 h, and 24 h exposure (Figure 2A). In contrast, after 1 h exposure animals in all three behavioral categories were observed. For the regular movement (RM) and NM variables, all exposure categories differed significantly from the control group (Supplementary Tables S2.1, S2.3), with lower proportions of animals in the RM group, and higher proportions in the NM category. No significant differences were found among the different exposure groups, but the *p*-values for comparisons of the NM category between the 1h and 6–24 h exposures were close to the significance level (Supplementary Table S2.3). For the irregular movement (IM) variable, the 1 h exposure group had significantly higher proportion of animals compared to all other exposure groups and to controls (Supplementary Table S2.2). In conclusion, 1 h exposure to reduced oxygen had mixed effect on the animals while 6–24 h exposure made all specimens immobile.

At the second check 10 h after exposure, the proportion of animals in the RM category tended to increase for all exposure groups, and in the 6 h and 12 h groups the proportion with normal movements was 97% and 87%, respectively, and did not differ statistically compared to the control group (Figure 2B; Supplementary Table S2.1). The proportion of RM animals in the 1 h, 18 h, and 24 h groups remained lower than the controls. There were also significantly higher proportions in the 6 h and 12 h groups compared to 18 h and 24 h, while the 1 h group did not differ significantly from 12 h, 18 h or 24 h. With the exception of the 1 h exposure, the proportion of NM animals tended to increase with longer exposure time, with the 24 h group having significantly higher values than the control, 6 h and 12 h groups (Supplementary Table S2.3). The 1 h and 18 h exposure groups showed a similar pattern that deviated from the other exposure groups, with considerable proportions of animals in the RM, IM, and NM groups.

### 3.2 Hypsibius exemplaris

**FIGURE 3:**
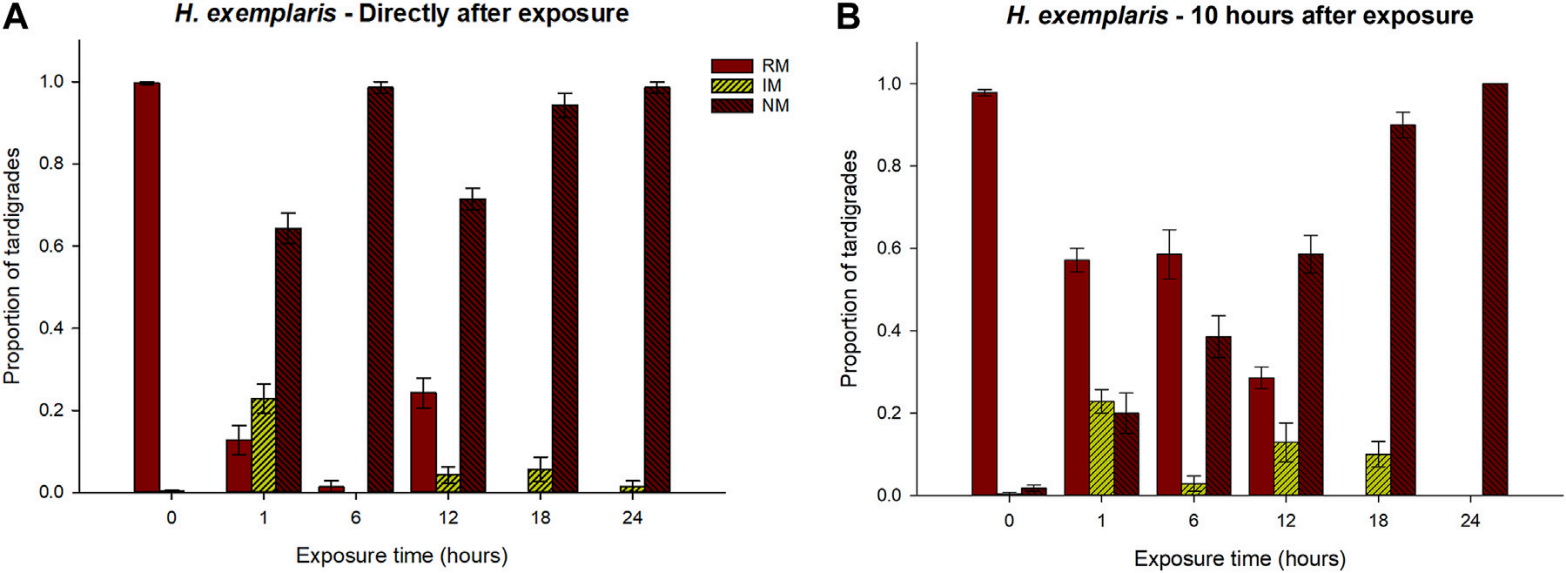
Responses of *H. exemplaris* to exposure of hypoxic conditions for different periods of time (0–24 h), in terms of proportions of tardigrades recorded in the three behavioral categories regular movement (RM), irregular movement (IM), and no movement (NM). 0 represents the control group that was not exposed to hypoxia. Panel **(A)** shows the results at the first check directly after exposure, while panel **(B)** shows the results 10 h after the exposure. Error bars represent 1 standard error from 7 replicate samples, each with 10 individual tardigrades. Statistical data for overall comparisons between exposure groups: Panel **(A)**. RM: χ^2^ = 59.2, *p* < 0.001. IM: χ^2^ = 38.3, *p* < 0.001. NM: χ^2^ = 60.0, *p* < 0.001. Panel **(B)**. RM: χ^2^ = 58.2, *p* < 0.001. IM: χ^2^ = 39.3, *p* < 0.001. NM: χ^2^ = 56.8, *p* < 0.001. Degrees of freedom = 5 in all comparisons.

Figure 3 and Supplementary Tables S2.4–S2.6 show the results for *H. exemplaris*. At the check directly after exposure, animals in a state of RM were observed in the 1 h, 6 h, and 12 h exposure groups, but with significantly lower proportions compared to the controls (Supplementary Table S2.4). The higher proportion of animals with RM at the 12 h exposure is also statistically different compared to the 18 h and 24 h categories, but not compared to the 1 h and 6 h categories.

For the NM category there were significant differences between the control group and all other exposure groups, with a higher proportion of tardigrades in an NM state in the exposure groups. The proportion of animals in the NM state was significantly lower in the 1 h group than in the 6 h and 24 h groups, while no statistical differences were found compared to the 12 h and 18 h exposure groups (Supplementary Table S2.6). Animals in the IM category were observed in all exposure groups, but with a significantly higher proportion in the 1 h group.

At the 10 h check, there were still no animals returning to a RM state after the 18 and 24 h exposures, while the proportion of animals in this state had increased in the 1 h, 6 h, and 12 h groups, but remaining significantly lower than the controls (Supplementary Table S2.4). In the 1 h and 6 h exposure groups, around 60% of the animals had returned to regular movements. The proportion of animals in the IM state did not show any dramatic change between the two checks but tended to be slightly higher at the 10 h check. The proportion of animals in the NM state tended to increase with increased exposure time, and in the 12–24 h group, most animals were in a state of no movements (Figure 3).

### 3.3 Comparison of responses to low oxygen in the two species

**FIGURE 4:**
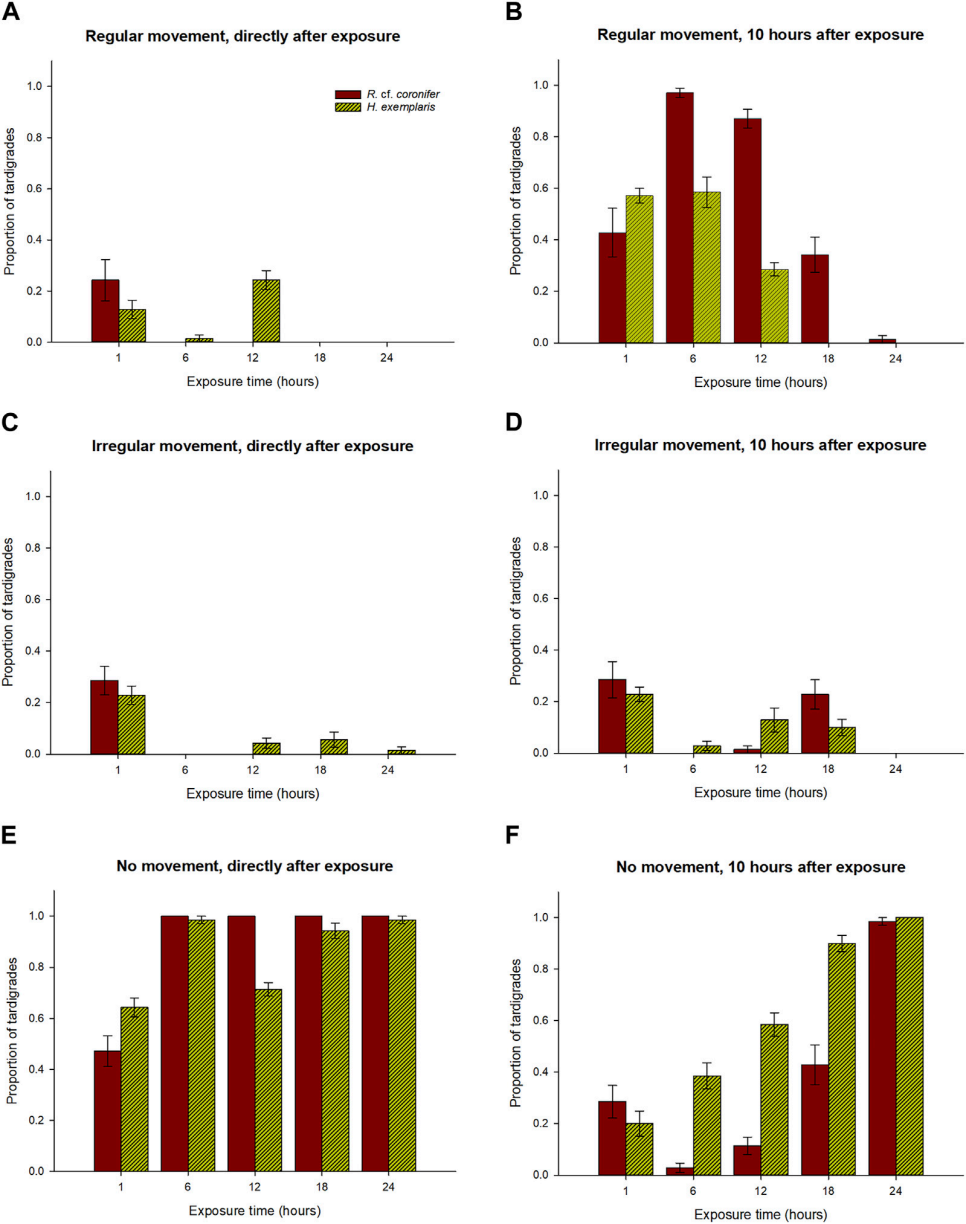
Comparisons between the two species *R.* cf. *coronifer* and *H. exemplaris* of the proportions of animals in each of the time exposure categories with respect to regular movement, irregular movement, and no movement. Panels **(A,C,E)** shows the results from the first check directly after exposure, while panels **(B,D,F)** shows the results 10 h after the exposure. Error bars represent 1 standard error from 7 replicate samples, each with 10 individual tardigrades. Note: this figure is based on the same data as in Figures 2, 3.

Figure 4 and Table 1 shows a comparison between the two species in their behavioral responses RM, IM, and NM to hypoxia, for the observations directly after exposure and after 10 h. At the first check the overall pattern was relatively similar for the two species, with most specimens for all exposure groups being in the NM group, thus strongly affected by the hypoxic conditions (Figures 4A, C, E). *H. exemplaris* had significantly higher proportion of RM animals in the 12 h exposure group, while *R.* cf. *coronifer* had higher proportions of NM animals at the 1 h exposure but lower proportions at the 12 h exposure.

**TABLE 1:** *p*-values in statistical comparisons (Kruskal–Wallis analysis) between *H. exemplaris* and *R.* cf. *coronifer* for the three behavioral responses regular, irregular and no movement, at the observations directly after exposure and 10 h after exposure. Values in bold indicate a significant difference (*p* < 0.05) between the species for a behavioral response and for a specific exposure category. Statistical data for the tests are given below the table. Degrees of freedom = 1 in all comparisons.

Directly after exposure	1 h	6 h	12 h	18 h	24 h
Regular movement	0.294	0.317	**0.001**	1.00	1.00
Irregular movement	0.178	1.000	0.060	0.061	0.317
No movement	**0.037**	0.317	**0.001**	0.061	0.317

10 h after exposure	1 h	6 h	12 h	18 h	24 h
Regular movement	0.260	**0.001**	**0.001**	**0.003**	0.317
Irregular movement	0.593	0.141	0.061	0.088	1.000
No movement	0.361	**0.001**	**0.001**	**0.002**	0.317

Directly after exposure. **1-h**: RM, χ^2^ = 1.1; IM, χ^2^ = 1.8; NM, χ^2^ = 4.4. **6-h**: RM, χ^2^ = 1.0; IM, χ^2^ = 0.0; NM, χ^2^ = 1.0. **12-h**: RM, χ^2^ = 11.3; IM, χ^2^ = 3.5; NM, χ^2^ = 11.5. **18-h**: RM, χ^2^ = 0.0; IM, χ^2^ = 3.5; NM, χ^2^ = 3.5. **24-h**: RM, χ^2^ = 0.0; IM, χ^2^ = 1.0; NM: χ^2^ = 1.0.

10 h after exposure. **1-h**: RM, χ^2^ = 1.3; IM, χ^2^ = 0.3; NM, χ^2^ = 0.8. **6-h**: RM, χ^2^ = 10.4; IM, χ^2^ = 2.2; NM, χ^2^ = 10.3. **12-h**: RM, χ^2^ = 10.3; IM, χ^2^ = 3.5; NM, χ^2^ = 10.5. **18-h**: RM, χ^2^ = 9.0; IM, χ^2^ = 2.9; NM, χ^2^ = 10.0. **24-h**: RM, χ^2^ = 1.0; IM, χ^2^ = 0.0; NM, χ^2^ = 1.0.

At the observations 10 h after exposure *R.* cf. *coronifer* had significantly higher proportions of animals in the RM state at the 6 h, 12 h, and 18 h exposures than *H. exemplaris*, suggesting that the latter species had a lower rate of recovery from hypoxia (Figures 4B, D, F). For the NM state the pattern was opposite, with significantly higher proportions of *H. exemplaris* in the 6 h, 12 h, and 18 h exposure groups. In the 24 h exposure all or almost all animals in both species were found in the NM state.

### 3.4 Effects of exposure to increased levels of hypoxia

**FIGURE 5:**
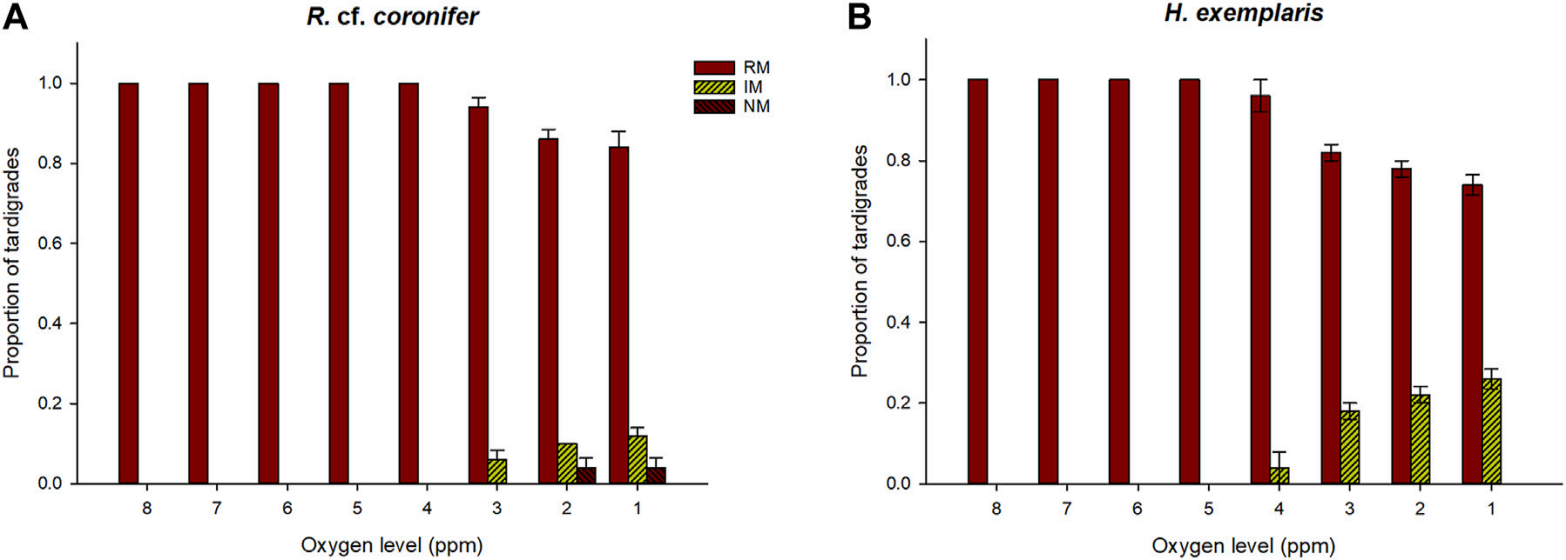
Behavioral responses to reductions in oxygen level, in terms of proportions of tardigrades recorded in the three behavioral categories regular movement (RM), irregular movement (IM), and no movement (NM). Panel **(A)** shows the results for *R.* cf. *coronifer* and panel **(B)** for *H. exemplaris*. Error bars represent 1 standard error from 5 replicate samples, each with 10 individual tardigrades.

Figure 5 shows that reduced levels of oxygen started to have an impact on the behavior of *R.* cf. *coronifer* at 3 ppm of oxygen, with some animals showing irregular movements, while in *H. exemplaris* such impact was first observed at 4 ppm. The proportion of animals with affected behavior increased with further reductions in oxygen levels, and more animals of *H. exemplaris* were affected than in *R.* cf. *coronifer*. No specimens of *H. exemplaris* entered an NM state, while some specimens of *R.* cf. *coronifer* did so, at the 1–2 ppm level.

The four lowest categories of oxygen were evaluated for differences between the species in responses to reduced oxygen levels. Table 2 shows that there was a significant difference between *H. exemplaris* and *R.* cf. *coronifer* at the 3 and 2 ppm levels of oxygen for the regular and irregular movement categories, with *H. exemplaris* being more affected by reduced oxygen than *R.* cf. *coronifer*, with lower proportion of animals in the RM state and higher proportion in the IM state. Also at the 1 ppm level, *H. exemplaris* had more animals in the IM state.

**TABLE 2:** *p*-values for statistical comparison between *R.* cf. *coronifer* and *H. exemplaris* in response to reduced oxygen levels, based on the data in Figure 5. RM = regular movement, IM = irregular movements, NM = no movement. Test statistics: **4 ppm: RM, χ^2^ = 1.0; IM, χ^2^ = 1.0; NM, χ^2^ = 0.0. 3 ppm: RM χ^2^ = 6.1; IM, χ^2^ = 6.1; NM, χ^2^ = 0.0. 2 ppm: RM χ^2^ = 4.1; IM, χ^2^ = 8.3; NM, χ^2^ = 2.3. 1 ppm: RM, χ^2^ = 3.1; IM, χ^2^ = 6.5; NM, χ^2^ = 2.3. Values in bold indicate statistically significant (*p* < 0.05) differences between the two species at the specific levels of oxygen. Degrees of freedom = 1 in all comparisons**.

Oxygen level (ppm)	RM	IM	NM
4	0.317	0.317	1
3	**0.014**	**0.014**	1
2	**0.042**	**0.004**	0.134
1	0.077	**0.011**	0.134

### 3.5 The rate of reoxygenation

**FIGURE 6:**
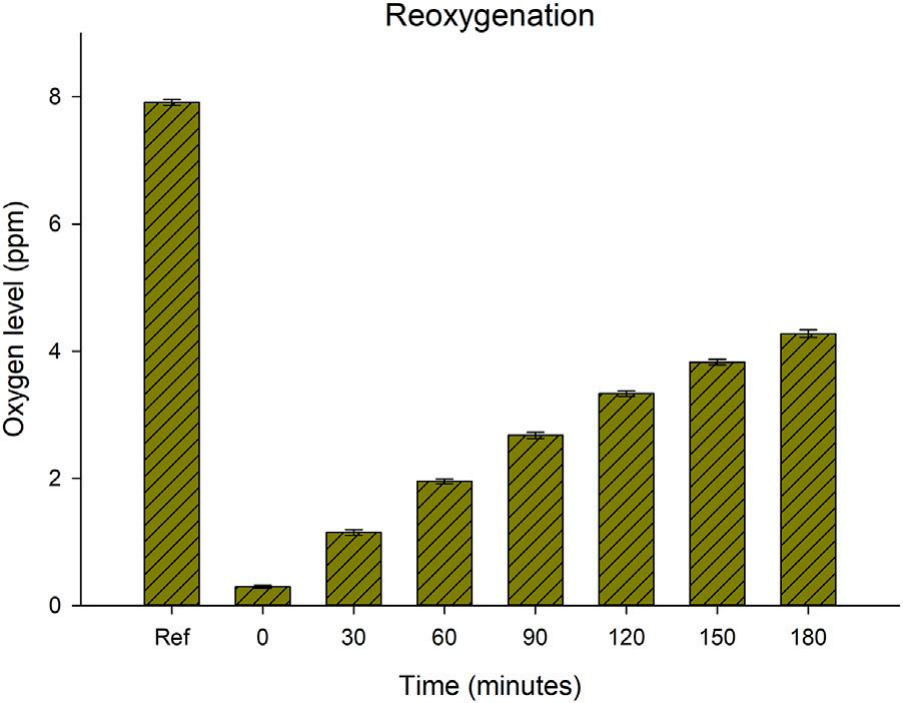
Reoxygenation over time in ambient laboratory conditions of 7 replicate samples of deionized water without tardigrades after reduction of oxygen by nitrogen purging. The first bar (“Ref”) represents the natural oxygen level before nitrogen purging (mean = 7.9 ppm, SD = 0.1), while the second bar (“0”) represents the oxygen level achieved after nitrogen purging for 40 min (mean = 0.3 ppm, SD = 0.05).

Figure 6 shows the rate of natural reoxygenation from the air after reducing oxygen level to on average 0.3 ppm. 30 min of exposure to air resulted in an increase in oxygen level to about 1 ppm, and after 60 min the oxygen levels had increased to about 2 ppm. At the last estimates the oxygen level had increased to 4.3 ppm, and the rate of reoxygenation declined to about 0.5 ppm per 30 min. Fitting a curve for the rate of reoxygenation and extrapolating from the curve function predicted that complete reoxygenation (8 ppm) would be reached after approx. 400 min (6.7 h) (y^2^ = a+bt^1.5^; y = oxygen level (ppm), *t* = time (min), a = 0.08756, b = 0.00791, *r* ^2^ = 0.998, TableCurve 2D v. 5.01).

## 4 Discussion

The results of this study show that tardigrades can recover activity after almost complete loss of free oxygen in their environment. However, in both *R*. cf. *coronifer* and *H. exemplaris* recovery generally declined with increased exposure time, with no or very few specimens recovering after 24 h exposure to hypoxia. In *R*. cf. *coronifer* a very high proportion of the animals returned to normal activity after 6 h and 12 h under hypoxic conditions. The fact that all of these animals were immobile directly after the exposure show that they had responded to the hypoxic conditions and entered the asphyctic state but were able to recover. The much lower proportion of full recovery after 1 h exposure is hard to explain, but many specimens in this exposure group were still active but showing irregular movements.

We did not distinguish between animals in a viable but asphyctic state and dead animals, and whether animals that were observed in an immobile state 10 h after the exposure were all dead, or if some of them were still viable but required more time to recover, remains to be evaluated in future studies. The data on reoxygenation showed that oxygen level reached 4 ppm within 3 h, a level at which little effect was seen on behavior in the experiment with sequential reduction of oxygen, and full reoxygenation was projected to be reached within 7 h. The immobile animals at the 10 h observation post-exposure therefore would have been exposed to about 7 h of oxygen conditions >4 ppm. However, increased time of recovery after long exposure to anoxia has indeed been reported in both *Caenorhabditis elegans* (Van Voorhies and Ward, 2000) and in embryos of *Artemia franciscana* (Clegg, 1997), and the relationship between exposure time to hypoxia and recovery time deserves investigation also in tardigrades. Positive correlations between recovery time and level of stress have previously been reported in tardigrades for exposure to desiccation [*rate of desiccation*, Horikawa and Higashi (2004); *time in dry state*; Crowe and Higgins (1967); Rebecchi et al. (2009)], probably representing periods of repair of cellular damage induced by the stress (Jönsson, 2003).

Both the exposure to different time periods of hypoxia and the response to gradually reduced oxygen levels indicate that *H. exemplaris* is slightly more sensitive to hypoxia than *R*. cf. *coronifer*. Additional studies should be made to confirm this indication of an interspecific difference, and also consider the possible evolutionary, ecological and physiological background to such difference. *H. exemplaris* is considered a freshwater tardigrade, and according to Nelson et al. (2015) certain aquatic tardigrade species can survive up to 3 days in the asphyctic state. Our study suggests much more limited tolerance under very low oxygen conditions, but it is possible that less severe hypoxic levels than used in our study allow better recovery of asphyctic tardigrades. Since the animals in our study started to respond to hypoxia already at 3–4 ppm, it would be interesting to investigate in future studies how the interaction between level of hypoxia and time of exposure affects the pattern of activity response and survival.

In comparison to the two other invertebrate phyla with many species showing cryptobiotic capability—nematodes and rotifers—the observed tolerance to hypoxia in *R*. cf. *coronifer* and *H. exemplaris* is relatively modest. Van Voorhies and Ward (2000) reported almost no mortality in adult *C. elegans* exposed to anoxia up to 48 h, 50% survival after 96 h, and no survival after 144 h. In another study by Kitazume et al. (2018) a large inter-specific difference in tolerance to hypoxia was reported in four nematode species, with one species (*Bursaphelenchus xylophilus*) showing >90% survival of adults after exposure to oxygen levels at < 0.01 ppm for 96 h. In rotifers, Ricci (2017) reported that 30% of the species *Macrotrachela quadricornifera* could survive in an anoxic environment for 6 days. The tolerance to hypoxia in tardigrades, nematodes and rotifers is however far behind embryos of the brine shrimp, *A*. *franciscana*, which has been reported to survive 4 years of continuous anoxic conditions in a hydrated state with assumed completely arrested metabolism (Clegg, 1997).

Since very few studies on hypoxia in tardigrades have been reported, the physiological mechanisms allowing tardigrades to survive under severe hypoxic conditions, and the factor(s) limiting the time that tardigrades can survive hypoxia, are unknown. An important question related to this is whether the animals enter an ametabolic state (anoxybiosis) in response to hypoxia, or are able to maintain a reduced metabolism under very low oxygen conditions. If metabolism is arrested, energetic constraints are unlikely to limit the time that animals can stay under hypoxic conditions and still recover. Instead, accumulation of damage on cellular components may then be the cause of the inability to recover after prolonged exposure to hypoxia. The relatively short time of exposure close to anoxic conditions that the tardigrade species investigated in this study were able to recover from could indicate that they do not tolerate a complete arrest of metabolism. Clearly, studies on the metabolic status of tardigrades exposed to hypoxia, and how the cells and tissues in these animals are affected by hypoxia, including “-omics” responses to hypoxia, are needed.

In many animals, an activation of endogenous antioxidant defenses connected with exposure to hypoxia has been documented, interpreted as a *preparation for oxidative stress* (POS) (Hermes-Lima et al., 1998; Hermes-Lima et al., 2015). Contrary to what was earlier believed, the hypoxic state or the exit from hypoxia may give rise to increased reactive oxygen species (ROS), counteracted by an increased antioxidant activity. The antioxidant defense system is considered to have a central role in the tolerance of tardigrades and other cryptobiotic invertebrates to desiccation and radiation (Rebecchi, 2013; Jönsson, 2019; Giovannini et al., 2022), and analyses of ROS generation and antioxidant responses connected with exposure to hypoxia in tardigrades would be of great interest. Also, the presence of hypoxia-inducible factor (HIF) genes (Graham and Presnell, 2017) are of interest for understanding hypoxia tolerance in tardigrades. Genes of the HIF family are highly conserved across metazoans, but loss of major HIF pathways has been documented within the crustacean group, also including species tolerating anoxia (Graham and Barreto, 2020). In several species of tardigrades (*Ramazzottius varieornatus, H. exemplaris, Paramacrobiotus* sp. *TYO, Echiniscus testudo*), representing both taxonomic classes (Heterotardigrada, Eutardigrada), loss of one of the major HIF transcriptional regulators, the HIF-1α pathway, has been reported (Hashimoto et al., 2016; Yoshida et al., 2017; Hara et al., 2021; Murai et al., 2021). This suggests that alternative mechanisms and genetic pathways for responses to hypoxic stress have evolved in tardigrades. Studies comparing antioxidant responses to exposure of desiccation, radiation and hypoxia would contribute to evaluation of the cross-tolerance hypothesis, suggesting a common defense mechanism behind tolerance to several environmental stresses in cryptobiotic invertebrates (Ryabova et al., 2017; Jönsson, 2019).

To the extent that resistance to hypoxia represents an adaptation to the environmental conditions experienced in the natural habitats of the two species, the present results do not suggest that *R.* cf. *coronifer* and *H. exemplaris* are naturally exposed to long-term hypoxia. *R.* cf. *coronifer* lives in mosses regularly exposed to desiccation, which may lead to temporary oxygen deficiency when the animal is captured in small water pockets in the process of dehydration of the moss. The duration of this condition will likely be short (minutes or hours), especially in the dry Alvar habitat where the population of *R.* cf. *coronifer* used in this study lives. *H. exemplaris* on the other hand lives in more permanently wet freshwater habitats which rarely dry up (Gąsiorek et al., 2018), but where hypoxic/anoxic levels may arise in the benthic layer due to high decomposition activity and low water circulation. In the context of hypoxia adaptations in tardigrades, the report by Kharkevych and Sergeeva (2013) of marine tardigrades living under permanent anoxic conditions in the Black Sea is of great interest, and more studies on the natural environmental conditions, hypoxia tolerance pattern, and metabolic system of these populations would be very valuable. A report by Kristensen and Hallas (1980) on two marine tardigrades of the genus *Echiniscoides* from Greenland inhabiting barnacle shells is also of interest. The animals were kept in a closed vial with decomposing barnacles for 6 months, presumably under anoxic conditions, and became active after aeration of the water. Future comparative studies on tolerance to hypoxia in tardigrade populations living in terrestrial, freshwater and marine ecosystems may reveal if there are general differences in tolerance, reflecting evolutionary adaptations to current environmental conditions or phylogenetic associations more related to the evolutionary history of different lineages.

## Data Availability

The original contributions presented in the study are included in the article/Supplementary Material, further inquiries can be directed to the corresponding author.
