# Supplementary material for: An experimental study on tolerance to hypoxia in tardigrades
Source: Front Physiol. 2023 Sep 5;14:1249773. doi: 10.3389/fphys.2023.1249773 (PMC10507709; doi:10.3389/fphys.2023.1249773)

Supplementary Material

An experimental study on tolerance to hypoxia in tardigrades

Philip Hagelbäck, K. Ingemar Jönsson*

*** Correspondence:** Corresponding Author: ingemar.jonsson@hkr.se

1 Supplementary Data

Supplementary Table S1. Original data on species, hypoxia treatment, which post-exposure check of animal behavior that data refer to (check 1 immediately after hypoxia exposure, or check 2 ten hours after exposure), and the proportion of animals in each replicate sample categorized into three behavioral categories; regular movement (RM), irregular movement (IM), and no movement (NM).

Species	Experimental category	Replicate sample	Post-exposure check	Proportion Regular movements (RM)	Proportion irregular movements (IM)	Proportion no movements (NM)
R. cf. coronifer	Treatment 1h	1	1	0.30	0.30	0.40
R. cf. coronifer	Treatment 1h	2	1	0.10	0.30	0.60
R. cf. coronifer	Treatment 1h	3	1	0.30	0.30	0.40
R. cf. coronifer	Treatment 1h	4	1	0.00	0.30	0.70
R. cf. coronifer	Treatment 1h	5	1	0.50	0.00	0.50
R. cf. coronifer	Treatment 1h	6	1	0.50	0.30	0.20
R. cf. coronifer	Treatment 1h	7	1	0.00	0.50	0.50
R. cf. coronifer	Treatment 1h	1	2	0.50	0.40	0.10
R. cf. coronifer	Treatment 1h	2	2	0.00	0.50	0.50
R. cf. coronifer	Treatment 1h	3	2	0.50	0.20	0.30
R. cf. coronifer	Treatment 1h	4	2	0.30	0.20	0.50
R. cf. coronifer	Treatment 1h	5	2	0.70	0.00	0.30
R. cf. coronifer	Treatment 1h	6	2	0.70	0.20	0.10
R. cf. coronifer	Treatment 1h	7	2	0.30	0.50	0.20
R. cf. coronifer	Treatment 6h	1	1	0.00	0.00	1.00
R. cf. coronifer	Treatment 6h	2	1	0.00	0.00	1.00

R. cf. coronifer	Treatment 6h	3	1	0.00	0.00	1.00
R. cf. coronifer	Treatment 6h	4	1	0.00	0.00	1.00
R. cf. coronifer	Treatment 6h	5	1	0.00	0.00	1.00
R. cf. coronifer	Treatment 6h	6	1	0.00	0.00	1.00
R. cf. coronifer	Treatment 6h	7	1	0.00	0.00	1.00
R. cf. coronifer	Control 6h	1	1	1.00	0.00	0.00
R. cf. coronifer	Control 6h	2	1	1.00	0.00	0.00
R. cf. coronifer	Control 6h	3	1	1.00	0.00	0.00
R. cf. coronifer	Control 6h	4	1	1.00	0.00	0.00
R. cf. coronifer	Control 6h	5	1	1.00	0.00	0.00
R. cf. coronifer	Control 6h	6	1	1.00	0.00	0.00
R. cf. coronifer	Control 6h	7	1	1.00	0.00	0.00
R. cf. coronifer	Treatment 6h	1	2	1.00	0.00	0.00
R. cf. coronifer	Treatment 6h	2	2	0.90	0.00	0.10
R. cf. coronifer	Treatment 6h	3	2	1.00	0.00	0.00
R. cf. coronifer	Treatment 6h	4	2	0.90	0.00	0.10
R. cf. coronifer	Treatment 6h	5	2	1.00	0.00	0.00
R. cf. coronifer	Treatment 6h	6	2	1.00	0.00	0.00
R. cf. coronifer	Treatment 6h	7	2	1.00	0.00	0.00
R. cf. coronifer	Control 6h	1	2	1.00	0.00	0.00
R. cf. coronifer	Control 6h	2	2	1.00	0.00	0.00
R. cf. coronifer	Control 6h	3	2	1.00	0.00	0.00
R. cf. coronifer	Control 6h	4	2	1.00	0.00	0.00
R. cf. coronifer	Control 6h	5	2	1.00	0.00	0.00
R. cf. coronifer	Control 6h	6	2	1.00	0.00	0.00
R. cf. coronifer	Control 6h	7	2	1.00	0.00	0.00
R. cf. coronifer	Treatment 12h	1	1	0.00	0.00	1.00
R. cf. coronifer	Treatment 12h	2	1	0.00	0.00	1.00
R. cf. coronifer	Treatment 12h	3	1	0.00	0.00	1.00
R. cf. coronifer	Treatment 12h	4	1	0.00	0.00	1.00
R. cf. coronifer	Treatment 12h	5	1	0.00	0.00	1.00
R. cf. coronifer	Treatment 12h	6	1	0.00	0.00	1.00
R. cf. coronifer	Treatment 12h	7	1	0.00	0.00	1.00
R. cf. coronifer	Control 12h	1	1	1.00	0.00	0.00
R. cf. coronifer	Control 12h	2	1	1.00	0.00	0.00
R. cf. coronifer	Control 12h	3	1	1.00	0.00	0.00
R. cf. coronifer	Control 12h	4	1	1.00	0.00	0.00
R. cf. coronifer	Control 12h	5	1	0.90	0.00	0.10
R. cf. coronifer	Control 12h	6	1	1.00	0.00	0.00
R. cf. coronifer	Control 12h	7	1	0.80	0.00	0.20
R. cf. coronifer	Treatment 12h	1	2	0.70	0.00	0.30
R. cf. coronifer	Treatment 12h	2	2	0.90	0.00	0.10
R. cf. coronifer	Treatment 12h	3	2	1.00	0.00	0.00
R. cf. coronifer	Treatment 12h	4	2	0.90	0.00	0.10
R. cf. coronifer	Treatment 12h	5	2	0.90	0.00	0.10
R. cf. coronifer	Treatment 12h	6	2	0.90	0.00	0.10

R. cf. coronifer	Treatment 12h	7	2	0.80	0.10	0.10
R. cf. coronifer	Control 12h	1	2	1.00	0.00	0.00
R. cf. coronifer	Control 12h	2	2	1.00	0.00	0.00
R. cf. coronifer	Control 12h	3	2	1.00	0.00	0.00
R. cf. coronifer	Control 12h	4	2	1.00	0.00	0.00
R. cf. coronifer	Control 12h	5	2	0.90	0.00	0.10
R. cf. coronifer	Control 12h	6	2	1.00	0.00	0.00
R. cf. coronifer	Control 12h	7	2	0.80	0.00	0.20
R. cf. coronifer	Treatment 18h	1	1	0.00	0.00	1.00
R. cf. coronifer	Treatment 18h	2	1	0.00	0.00	1.00
R. cf. coronifer	Treatment 18h	3	1	0.00	0.00	1.00
R. cf. coronifer	Treatment 18h	4	1	0.00	0.00	1.00
R. cf. coronifer	Treatment 18h	5	1	0.00	0.00	1.00
R. cf. coronifer	Treatment 18h	6	1	0.00	0.00	1.00
R. cf. coronifer	Treatment 18h	7	1	0.00	0.00	1.00
R. cf. coronifer	Control 18h	1	1	1.00	0.00	0.00
R. cf. coronifer	Control 18h	2	1	1.00	0.00	0.00
R. cf. coronifer	Control 18h	3	1	0.80	0.00	0.20
R. cf. coronifer	Control 18h	4	1	0.90	0.00	0.10
R. cf. coronifer	Control 18h	5	1	1.00	0.00	0.00
R. cf. coronifer	Control 18h	6	1	1.00	0.00	0.00
R. cf. coronifer	Control 18h	7	1	1.00	0.00	0.00
R. cf. coronifer	Treatment 18h	1	2	0.60	0.30	0.10
R. cf. coronifer	Treatment 18h	2	2	0.40	0.20	0.40
R. cf. coronifer	Treatment 18h	3	2	0.40	0.40	0.20
R. cf. coronifer	Treatment 18h	4	2	0.30	0.10	0.60
R. cf. coronifer	Treatment 18h	5	2	0.00	0.40	0.60
R. cf. coronifer	Treatment 18h	6	2	0.40	0.00	0.60
R. cf. coronifer	Treatment 18h	7	2	0.30	0.20	0.50
R. cf. coronifer	Control 18h	1	2	1.00	0.00	0.00
R. cf. coronifer	Control 18h	2	2	1.00	0.00	0.00
R. cf. coronifer	Control 18h	3	2	0.80	0.00	0.20
R. cf. coronifer	Control 18h	4	2	0.90	0.00	0.10
R. cf. coronifer	Control 18h	5	2	1.00	0.00	0.00
R. cf. coronifer	Control 18h	6	2	1.00	0.00	0.00
R. cf. coronifer	Control 18h	7	2	1.00	0.00	0.00
R. cf. coronifer	Treatnet 24h	1	1	0.00	0.00	1.00
R. cf. coronifer	Treatnet 24h	2	1	0.00	0.00	1.00
R. cf. coronifer	Treatnet 24h	3	1	0.00	0.00	1.00
R. cf. coronifer	Treatnet 24h	4	1	0.00	0.00	1.00
R. cf. coronifer	Treatnet 24h	5	1	0.00	0.00	1.00
R. cf. coronifer	Treatnet 24h	6	1	0.00	0.00	1.00
R. cf. coronifer	Treatnet 24h	7	1	0.00	0.00	1.00
R. cf. coronifer	Control 24h	1	1	1.00	0.00	0.00
R. cf. coronifer	Control 24h	2	1	1.00	0.00	0.00
R. cf. coronifer	Control 24h	3	1	1.00	0.00	0.00

R. cf. coronifer	Control 24h	4	1	1.00	0.00	0.00
R. cf. coronifer	Control 24h	5	1	1.00	0.00	0.00
R. cf. coronifer	Control 24h	6	1	1.00	0.00	0.00
R. cf. coronifer	Control 24h	7	1	1.00	0.00	0.00
R. cf. coronifer	Treatnet 24h	1	2	0.10	0.00	0.90
R. cf. coronifer	Treatnet 24h	2	2	0.00	0.00	1.00
R. cf. coronifer	Treatnet 24h	3	2	0.00	0.00	1.00
R. cf. coronifer	Treatnet 24h	4	2	0.00	0.00	1.00
R. cf. coronifer	Treatnet 24h	5	2	0.00	0.00	1.00
R. cf. coronifer	Treatnet 24h	6	2	0.00	0.00	1.00
R. cf. coronifer	Treatnet 24h	7	2	0.00	0.00	1.00
R. cf. coronifer	Control 24h	1	2	0.90	0.00	0.10
R. cf. coronifer	Control 24h	2	2	0.90	0.00	0.10
R. cf. coronifer	Control 24h	3	2	1.00	0.00	0.00
R. cf. coronifer	Control 24h	4	2	0.80	0.00	0.20
R. cf. coronifer	Control 24h	5	2	0.90	0.00	0.10
R. cf. coronifer	Control 24h	6	2	0.90	0.00	0.10
R. cf. coronifer	Control 24h	7	2	0.70	0.00	0.30
H. exemplaris	Treatment 1h	1	1	0.20	0.10	0.70
H. exemplaris	Treatment 1h	2	1	0.20	0.30	0.50
H. exemplaris	Treatment 1h	3	1	0.20	0.20	0.60
H. exemplaris	Treatment 1h	4	1	0.20	0.20	0.60
H. exemplaris	Treatment 1h	5	1	0.10	0.20	0.70
H. exemplaris	Treatment 1h	6	1	0.00	0.20	0.80
H. exemplaris	Treatment 1h	7	1	0.00	0.40	0.60
H. exemplaris	Treatment 1h	1	2	0.50	0.20	0.30
H. exemplaris	Treatment 1h	2	2	0.60	0.30	0.10
H. exemplaris	Treatment 1h	3	2	0.60	0.20	0.20
H. exemplaris	Treatment 1h	4	2	0.50	0.30	0.20
H. exemplaris	Treatment 1h	5	2	0.70	0.30	0.00
H. exemplaris	Treatment 1h	6	2	0.60	0.20	0.20
H. exemplaris	Treatment 1h	7	2	0.50	0.10	0.40
H. exemplaris	Treatment 6h	1	1	0.00	0.00	1.00
H. exemplaris	Treatment 6h	2	1	0.00	0.00	1.00
H. exemplaris	Treatment 6h	3	1	0.00	0.00	1.00
H. exemplaris	Treatment 6h	4	1	0.10	0.00	0.90
H. exemplaris	Treatment 6h	5	1	0.00	0.00	1.00
H. exemplaris	Treatment 6h	6	1	0.00	0.00	1.00
H. exemplaris	Treatment 6h	7	1	0.00	0.00	1.00
H. exemplaris	Control 6h	1	1	1.00	0.00	0.00
H. exemplaris	Control 6h	2	1	1.00	0.00	0.00
H. exemplaris	Control 6h	3	1	1.00	0.00	0.00
H. exemplaris	Control 6h	4	1	1.00	0.00	0.00
H. exemplaris	Control 6h	5	1	1.00	0.00	0.00
H. exemplaris	Control 6h	6	1	1.00	0.00	0.00
H. exemplaris	Control 6h	7	1	1.00	0.00	0.00

H. exemplaris	Treatment 6h	1	2	0.60	0.00	0.40
H. exemplaris	Treatment 6h	2	2	0.80	0.00	0.20
H. exemplaris	Treatment 6h	3	2	0.60	0.10	0.30
H. exemplaris	Treatment 6h	4	2	0.70	0.00	0.30
H. exemplaris	Treatment 6h	5	2	0.30	0.10	0.60
H. exemplaris	Treatment 6h	6	2	0.50	0.00	0.50
H. exemplaris	Treatment 6h	7	2	0.60	0.00	0.40
H. exemplaris	Control 6h	1	2	1.00	0.00	0.00
H. exemplaris	Control 6h	2	2	1.00	0.00	0.00
H. exemplaris	Control 6h	3	2	1.00	0.00	0.00
H. exemplaris	Control 6h	4	2	0.90	0.00	0.10
H. exemplaris	Control 6h	5	2	1.00	0.00	0.00
H. exemplaris	Control 6h	6	2	0.90	0.00	0.10
H. exemplaris	Control 6h	7	2	1.00	0.00	0.00
H. exemplaris	Treatment 12h	1	1	0.30	0.00	0.70
H. exemplaris	Treatment 12h	2	1	0.20	0.00	0.80
H. exemplaris	Treatment 12h	3	1	0.20	0.10	0.70
H. exemplaris	Treatment 12h	4	1	0.30	0.00	0.70
H. exemplaris	Treatment 12h	5	1	0.40	0.00	0.60
H. exemplaris	Treatment 12h	6	1	0.10	0.10	0.80
H. exemplaris	Treatment 12h	7	1	0.20	0.10	0.70
H. exemplaris	Control 12h	1	1	1.00	0.00	0.00
H. exemplaris	Control 12h	2	1	1.00	0.00	0.00
H. exemplaris	Control 12h	3	1	1.00	0.00	0.00
H. exemplaris	Control 12h	4	1	1.00	0.00	0.00
H. exemplaris	Control 12h	5	1	1.00	0.00	0.00
H. exemplaris	Control 12h	6	1	1.00	0.00	0.00
H. exemplaris	Control 12h	7	1	1.00	0.00	0.00
H. exemplaris	Treatment 12h	1	2	0.20	0.30	0.50
H. exemplaris	Treatment 12h	2	2	0.30	0.00	0.70
H. exemplaris	Treatment 12h	3	2	0.30	0.20	0.50
H. exemplaris	Treatment 12h	4	2	0.40	0.00	0.60
H. exemplaris	Treatment 12h	5	2	0.30	0.20	0.50
H. exemplaris	Treatment 12h	6	2	0.30	0.20	0.50
H. exemplaris	Treatment 12h	7	2	0.20	0.00	0.80
H. exemplaris	Control 12h	1	2	1.00	0.00	0.00
H. exemplaris	Control 12h	2	2	1.00	0.00	0.00
H. exemplaris	Control 12h	3	2	1.00	0.00	0.00
H. exemplaris	Control 12h	4	2	1.00	0.00	0.00
H. exemplaris	Control 12h	5	2	1.00	0.00	0.00
H. exemplaris	Control 12h	6	2	0.90	0.00	0.10
H. exemplaris	Control 12h	7	2	1.00	0.00	0.00
H. exemplaris	Treatment 18h	1	1	0.00	0.20	0.80
H. exemplaris	Treatment 18h	2	1	0.00	0.10	0.90
H. exemplaris	Treatment 18h	3	1	0.00	0.00	1.00
H. exemplaris	Treatment 18h	4	1	0.00	0.00	1.00

H. exemplaris	Treatment 18h	5	1	0.00	0.00	1.00
H. exemplaris	Treatment 18h	6	1	0.00	0.10	0.90
H. exemplaris	Treatment 18h	7	1	0.00	0.00	1.00
H. exemplaris	Control 18h	1	1	1.00	0.00	0.00
H. exemplaris	Control 18h	2	1	1.00	0.00	0.00
H. exemplaris	Control 18h	3	1	1.00	0.00	0.00
H. exemplaris	Control 18h	4	1	1.00	0.00	0.00
H. exemplaris	Control 18h	5	1	1.00	0.00	0.00
H. exemplaris	Control 18h	6	1	0.90	0.10	0.00
H. exemplaris	Control 18h	7	1	1.00	0.00	0.00
H. exemplaris	Treatment 18h	1	2	0.00	0.20	0.80
H. exemplaris	Treatment 18h	2	2	0.00	0.10	0.90
H. exemplaris	Treatment 18h	3	2	0.00	0.10	0.90
H. exemplaris	Treatment 18h	4	2	0.00	0.00	1.00
H. exemplaris	Treatment 18h	5	2	0.00	0.00	1.00
H. exemplaris	Treatment 18h	6	2	0.00	0.20	0.80
H. exemplaris	Treatment 18h	7	2	0.00	0.10	0.90
H. exemplaris	Control 18h	1	2	1.00	0.00	0.00
H. exemplaris	Control 18h	2	2	1.00	0.00	0.00
H. exemplaris	Control 18h	3	2	1.00	0.00	0.00
H. exemplaris	Control 18h	4	2	1.00	0.00	0.00
H. exemplaris	Control 18h	5	2	1.00	0.00	0.00
H. exemplaris	Control 18h	6	2	0.90	0.10	0.00
H. exemplaris	Control 18h	7	2	1.00	0.00	0.00
H. exemplaris	Treatment 24h	1	1	0.00	0.00	1.00
H. exemplaris	Treatment 24h	2	1	0.00	0.00	1.00
H. exemplaris	Treatment 24h	3	1	0.00	0.00	1.00
H. exemplaris	Treatment 24h	4	1	0.00	0.00	1.00
H. exemplaris	Treatment 24h	5	1	0.00	0.10	0.90
H. exemplaris	Treatment 24h	6	1	0.00	0.00	1.00
H. exemplaris	Treatment 24h	7	1	0.00	0.00	1.00
H. exemplaris	Control 24h	1	1	1.00	0.00	0.00
H. exemplaris	Control 24h	2	1	1.00	0.00	0.00
H. exemplaris	Control 24h	3	1	1.00	0.00	0.00
H. exemplaris	Control 24h	4	1	1.00	0.00	0.00
H. exemplaris	Control 24h	5	1	1.00	0.00	0.00
H. exemplaris	Control 24h	6	1	1.00	0.00	0.00
H. exemplaris	Control 24h	7	1	1.00	0.00	0.00
H. exemplaris	Treatment 24h	1	2	0.00	0.00	1.00
H. exemplaris	Treatment 24h	2	2	0.00	0.00	1.00
H. exemplaris	Treatment 24h	3	2	0.00	0.00	1.00
H. exemplaris	Treatment 24h	4	2	0.00	0.00	1.00
H. exemplaris	Treatment 24h	5	2	0.00	0.00	1.00
H. exemplaris	Treatment 24h	6	2	0.00	0.00	1.00
H. exemplaris	Treatment 24h	7	2	0.00	0.00	1.00
H. exemplaris	Control 24h	1	2	1.00	0.00	0.00

H. exemplaris	Control 24h	2	2	1.00	0.00	0.00
H. exemplaris	Control 24h	3	2	1.00	0.00	0.00
H. exemplaris	Control 24h	4	2	1.00	0.00	0.00
H. exemplaris	Control 24h	5	2	1.00	0.00	0.00
H. exemplaris	Control 24h	6	2	0.90	0.00	0.10
H. exemplaris	Control 24h	7	2	0.90	0.00	0.10

2 Supplementary Tables

Supplementary Table S2. Statistical results (P-values) from pairwise comparisons between hypoxia exposure groups for proportions of animals in different activity categories (RM, IM, NM) and at different times of post-exposure check (direct check, 10h check), using Kruskal-Wallis Analysis of Variance. P-values < 0.05 was considered statistically significant.

Table S2.1. Statistical data for *R.* cf. *coronifer* for the regular movement (RM) category. The uncorrected P-values was used as the base in the study. The adjusted P-values (Bonferroni) are presented in parenthesis. Significant values are marked red/bold. A significant value indicates a difference between the two exposure groups.

**Direct Check**	**Control**	**1 hour**	**6 hours**	**12 hours**	**18 hours**	**24 hours**
Control		**0.002 (0.036)**	**0.000 (0.000)**	**0.000 (0.000)**	**0.000 (0.000)**	**0.000 (0.000)**
1 hour			0.163 (1.000)	0.163 (1.000)	0.163 (1.000)	0.163 (1.000)
6 hours				1.000 (1.000)	1.000 (1.000)	1.000 (1.000)
12 hours					1.000 (1.000)	1.000 (1.000)
18 hours						1.000 (1.000)
24 hours						

**10 h Check**	**Control**	**1 hour**	**6 hours**	**12 hours**	**18 hours**	**24 hours**
Control		**0.001 (0.002)**	0.731 (1.000)	0.158 (1.000)	**0.000 (0.001)**	**0.000 (0.000)**
1 hour			**0.001 (0.014)**	0.056 (0.833)	0.833 (1.000)	0.295 (1.000)
6 hours				0.165 (1.000)	**0.000 (0.007)**	**0.000 (0.000)**
12 hours					**0.034** (0.503)	**0.003 (0.046)**
18 hours						0.403 (1.000)
24 hours						

Table S2.2. Statistical data for *R.* cf. *coronifer* for the regular movement (IM) category. The uncorrected P-values was used as the base in the study. The adjusted P-values (Bonferroni) are presented in parenthesis. Significant values are marked red/bold. A significant value indicates a difference between the two exposure groups.

Direct Check	Control	1 hour	6 hours	12 hours	18 hours	24 hours
Control		**0.000 (0.000)**	1.000 (1.000)	1.000 (1.000)	1.000 (1.000)	1.000 (1.000)
1 hour			**0.000 (0.000)**	**0.000 (0.000)**	**0.000 (0.000)**	**0.000 (0.000)**
6 hours				1.000 (1.000)	1.000 (1.000)	1.000 (1.000)
12 hours					1.000 (1.000)	1.000 (1.000)
18 hours						1.000 (1.000)
24 hours						

10 h Check	Control	1 hour	6 hours	12 hours	18 hours	24 hours
Control		**0.000 (0.000)**	1.000 (1.000)	0.497 (1.000)	**0.000 (0.000)**	1.000 (1.000)
1 hour			**0.000 (0.001)**	**0.000 (0.006)**	0.829 (1.000)	**0.000 (0.001)**
6 hours				0.592 (1.000)	**0.000 (0.002)**	1.000 (1.000)
12 hours					**0.001 (0.014)**	0.592 (1.000)
18 hours						**0.000 (0.002)**
24 hours						

Table S.2.3 Statistical data for *R.* cf. *coronifer* for the regular movement (NM) category. The uncorrected P-values was used as the base in the study. The adjusted P-values (Bonferroni) are presented in parenthesis. Significant values are marked red/bold. A significant value indicates a difference between the two exposure groups.

**Direct Check**	**Control**	**1 hour**	**6 hours**	**12 hours**	**18 hours**	**24 hours**
Control		**0.016 (0.24)**	**0.000 (0.000)**	**0.000 (0.000)**	**0.000 (0.000)**	**0.000 (0.000)**
1 hour			0.052 (0.78)	0.052 (0.78)	0.052 (0.78)	0.052 (0.78)
6 hours				1.000 (1.000)	1.000 (1.000)	1.000 (1.000)
12 hours					1.000 (1.000)	1.000 (1.000)
18 hours						1.000 (1.000)
24 hours						

**10 h Check**	**Control**	**1 hour**	**6 hours**	**12 hours**	**18 hours**	**24 hours**
Control		**0.002 (0.037)**	0.68 (1.000)	0.16 (1.000)	**0.000 (0.003)**	**0.000 (0.000)**
1 hour			**0.007** (0.098)	0.20 (1.000)	0.59 (1.000)	0.087 (1.000)
6 hours				0.15 (1.000)	**0.001 (0.017)**	**0.000 (0.000)**
12 hours					0.069 (1.000)	**0.003 (0.042)**
18 hours						0.24 (1.000)
24 hours						

Table S2.4. Statistical data for *H. exemplaris* for the regular movement (RM) category. The uncorrected P-values was used as the base in the study. The adjusted P-values (Bonferroni) are presented in parenthesis. Significant values are marked red/bold. A significant value indicates a difference between the two exposure groups.

**Direct Check**	**Control**	**1 hour**	**6 hours**	**12 hours**	**18 hours**	**24 hours**
Control		**0.000 (0.005)**	**0.000 (0.000)**	**0.009** (0.129)	**0.000 (0.000)**	**0.000 (0.000)**
1 hour			0.276 (1.000)	0.436 (1.000)	0.199 (1.000)	0.199 (1.000)
6 hours				0.062 (0.927)	0.846 (1.000)	0.846 (1.000)
12 hours					**0.039** (0.587)	**0.039** (0.587)
18 hours						1.000 (1.000)
24 hours						

**10 h Check**	**Control**	**1 hour**	**6 hours**	**12 hours**	**18 hours**	**24 hours**
Control		**0.004** (0.062)	**0.006** (0.084)	**0.000 (0.001)**	**0.000 (0.000)**	**0.000 (0.000)**
1 hour			0.940 (1.000)	0.318 (1.000)	**0.032** (0.478)	**0.032** (0.478)
6 hours				0.283 (1.000)	**0.026** (0.395)	**0.026** (0.395)
12 hours					0.251 (1.000)	0.251 (1.000)
18 hours						1.000 (1.000)
24 hours						

Table S2.5. Statistical data for *H. exemplaris* for the regular movement (IM) category. The uncorrected P-values was used as the base in the study. The adjusted P-values (Bonferroni) are presented in parenthesis. Significant values are marked red/bold. A significant value indicates a difference between the two exposure groups.

**Direct Check**	**Control**	**1 hour**	**6 hours**	**12 hours**	**18 hours**	**24 hours**
Control		**0.000 (0.000)**	0.862 (1.000)	0.057 (0.085)	**0.039** (0.582)	0.603 (1.000)
1 hour			**0.000 (0.000)**	**0.002 (0.003)**	**0.003 (0.046)**	**0.000 (0.000)**
6 hours				0.100 (1.000)	0.077 (1.000)	0.584 (1.000)
12 hours					0.899 (1.000)	0.273 (1.000)
18 hours						0.221 (1.000)
24 hours						

**10 h Check**	**Control**	**1 hour**	**6 hours**	**12 hours**	**18 hours**	**24 hours**
Control		**0.000 (0.000)**	0.309 (1.000)	**0.003 (0.041)**	**0.002 (0.029)**	0.885 (1.000)
1 hour			**0.001 (0.009)**	0.062 (0.936)	0.075 (1.000)	**0.000 (0.000)**
6 hours				0.117 (1.000)	0.099 (1.000)	0.359 (1.000)
12 hours					0.935 (1.000)	**0.013** (0.195)
18 hours						**0.010** (0.154)
24 hours						

Table S2.6. Statistical data for *H. exemplaris* for the regular movement (NM) category. The uncorrected P-values was used as the base in the study. The adjusted P-values (Bonferroni) are presented in parenthesis. Significant values are marked red/bold. A significant value indicates a difference between the two exposure groups.

Direct Check	Control	1 hour	6 hours	12 hours	18 hours	24 hours
Control		**0.008** (0.117)	**0.000 (0.000)**	**0.002 (0.029)**	**0.000 (0.000)**	**0.000 (0.000)**
1 hour			**0.030** (0.455)	0.73 (1.000)	0.072 (1.000)	**0.030** (0.455)
6 hours				0.069 (1.000)	0.711 (1.000)	1.000 (1.000)
12 hours					0.147 (1.000)	0.069 (1.000)
18 hours						0.711 (1.000)
24 hours						

10 h Check	Control	1 hour	6 hours	12 hours	18 hours	24 hours
Control		**0.043** (0.649)	**0.002 (0.024)**	**0.000 (0.001)**	**0.000 (0.000)**	**0.000 (0.000)**
1 hour			0.371 (1.000)	0.118 (1.000)	**0.013** (0.20)	**0.003 (0.039)**
6 hours				0.503 (1.000)	**0.114** (1.000)	**0.034** (0.509)
12 hours					0.363 (1.000)	0.147 (1.000)
18 hours						0.588 (1.000)
24 hours						

3 Supplementary Figures

Supplementary Figure S3. Photos showing how the 100 ml Duran® laboratory glass bottles were serially connected for exposing tardigrades to hypoxic conditions by means of a nitrogen gas flow through the plastic tubes (upper panel). The lower panels show the rubber stopper with double tubing (left) and the aquarium diffusion stone (right). Photos: Philip Hagelbäck.

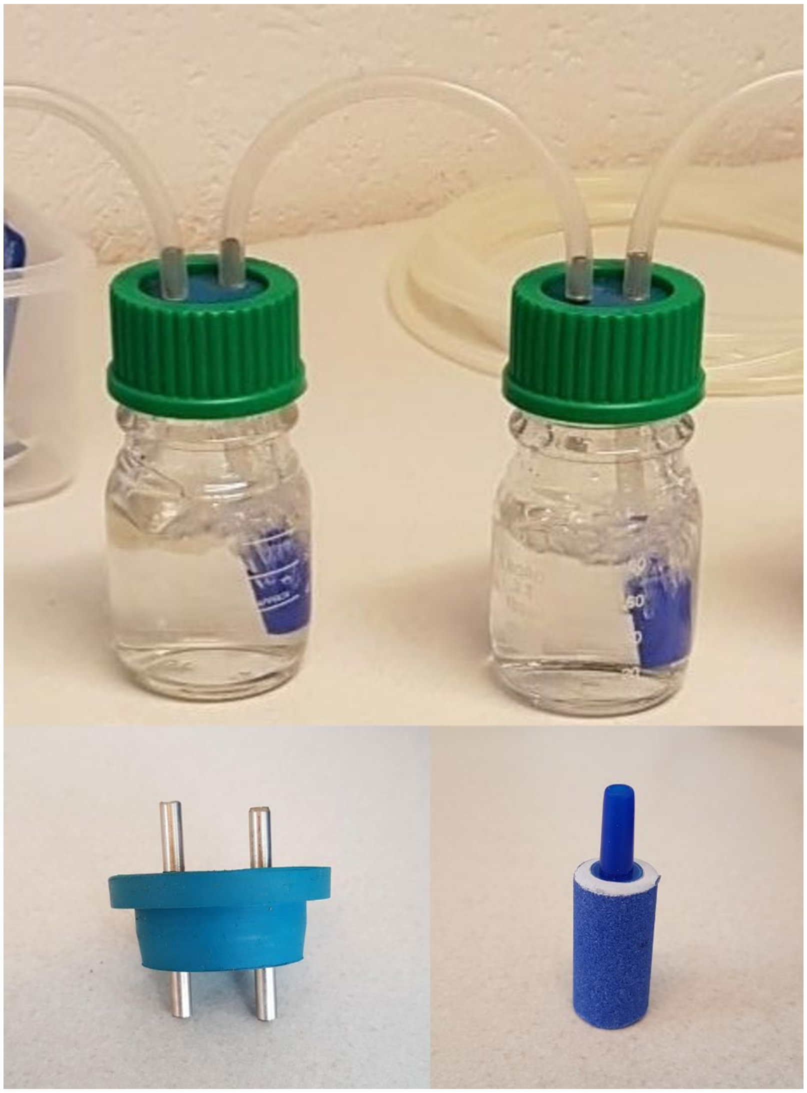

Supplement: Supplementary file 1 [file DataSheet1.PDF]
